# Lupus Nephritis Disguised: The Diagnostic Challenge of Eosinophilic Enteritis - A Case Report

**DOI:** 10.34172/mejdd.2024.372

**Published:** 2024-01-31

**Authors:** Chetan Phadke, Atul Sajgure, Charan Bale, Pavan Wakhare, Nilesh Shinde, Abhijit Chavan, Akshay Kulkarni, Shreeharsh Godbole, Anuja Makan, Debapriya Saha, Tushar Dighe

**Affiliations:** ^1^Department of Nephrology, Dr D. Y. Patil Medical College, Hospital and Research Centre, Pimpri, Pune, India; ^2^Nephrology Services, Dr D. Y. Patil Medical College, Hospital and Research Centre, Pimpri, Pune, India

**Keywords:** Lupus nephritis, Eosinophilic enteritis, Glomerulonephritis, SLE

## Abstract

Systemic lupus erythematosus (SLE) is a multi-systemic disorder affecting almost all systems of the body. Involvement of the kidney in this condition is known as lupus nephritis (LN). LN is one of the important disease manifestations of SLE with considerable influence on patient outcomes in terms of morbidity and mortality. A 33-year-old female came to the OPD with complaints of abdominal pain, infrequent loose stools since 4 months. The patient also had joint pain, predominantly small joints, since 2 months. Patient was admitted and all routine investigations were done. Patient underwent an oesophagogastroduodenoscopy (OGD) and colonoscopy for her abdominal pain and loose stools which did not respond to routine medication. Grossly there was edema present in the oesophagus and colon which on microscopy showed eosinophilic infiltration. Urine routine of the patient showed protein 1+and 24-hour urine protein quantification of 1427 mg/24 h. On further evaluation patient was found to have a positive ANA blot (dsDNA, AMAM2, Ro52 and Sm). The patient was planned for a renal biopsy in view of the proteinuria and positive ANA blot. The patient underwent a renal biopsy under USG guidance and was found to have Lupus nephritis Class 3 (ISN RPS staging). SLE is a multi-organ involving disease which if not diagnosed at the earliest can have serious complications and lead to end stage organ failure and even death. Atypical presentations often pose a diagnostic dilemma and may delay diagnosis and treatment. Early diagnosis and treatment can give patients of SLE a long and normal life. Diagnostic guidelines have helped in the diagnosis of such atypical presentations.

## Introduction

 Systemic lupus erythematosus (SLE) is a multi-systemic disorder affecting almost all systems of the body. Involvement of the kidney in this condition is known as lupus nephritis (LN). LN is one of the important disease manifestations of SLE, with considerable influence on patient’s outcomes in terms of morbidity and mortality.^1^ It is prevalent among young women with a peak age of onset between the late teens and early 40s and a female-to-male ratio of 9:1.^2^ The etiology of SLE is multi-factorial, including genetic, hormonal, and environmental factors. In most patients, SLE manifests chiefly through hematological, renal, and cerebral manifestations. If left untreated or if the diagnosis is missed, its complications can often be fatal. The fatal complications include lupus cerebritis, LN, and cardiac manifestations such as pericardial effusion.

## Case Report

 A 33-year-old woman came to the out-patient department with complaints of abdominal pain, and infrequent loose stools for 4 months. The patient also had joint pain, predominantly in small joints, for 2 months. She did not have any urinary complaints of dysuria or any abnormal discolouration of urine. The patient was admitted to the wards for investigation of the intestinal symptoms. On presentation, vitals were well within normal limits: pulse rate of 98/m, blood pressure (BP) of 128/74 mm Hg, and oxygen saturation of 99% in room air. Systemic examination revealed only mild generalized tenderness all over the abdomen. Symptomatic treatment was initially started for the patient with injectable pantoprazole 40 mg once a day, injectable ondansetron 4 mg thrice a day, injectable metronidazole 500 mg thrice a day and injectable hyoscine butyl bromide as required for pain relief. The patient’s symptoms did not respond to the intravenous therapy after 72 hours. Following were the routine lab investigations:

 Hb: 11.7 gm%, Total leucocyte count: 8800/cumm, platelet count 2.21 lakh/cumm, urea 18 mg/dL, creatinine 0.45 mg/dL, Na 137 mmol/L, K 3.9 mmol/L, urine routine showed Prot 1 + , RBC 3-4/ hpf, Pus cells: 1-2/hpf, Urine protein creatinine ratio: 3.06 mg/mg.

 The patient underwent abdominal ultrasonography, which showed no significant abnormality of the bowel loops with normal-sized kidneys, only with a mild increase in echogenicity. Upper gastrointestinal (UGI) endoscopy and colonoscopy were planned for this patient in view of persistent symptoms. A nephrologist’s opinion was sought in view of the unexplained urinary protein. While the UGI endoscopy was done, a 24-hour urine protein collection was advised.

**Figure 1:**
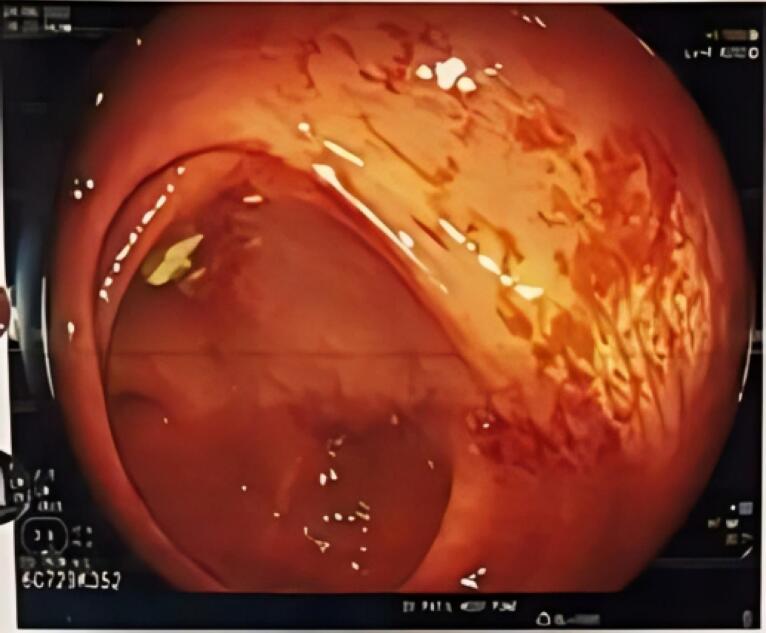
Edematous bowel loop on colonoscopy

**Figure 2:**
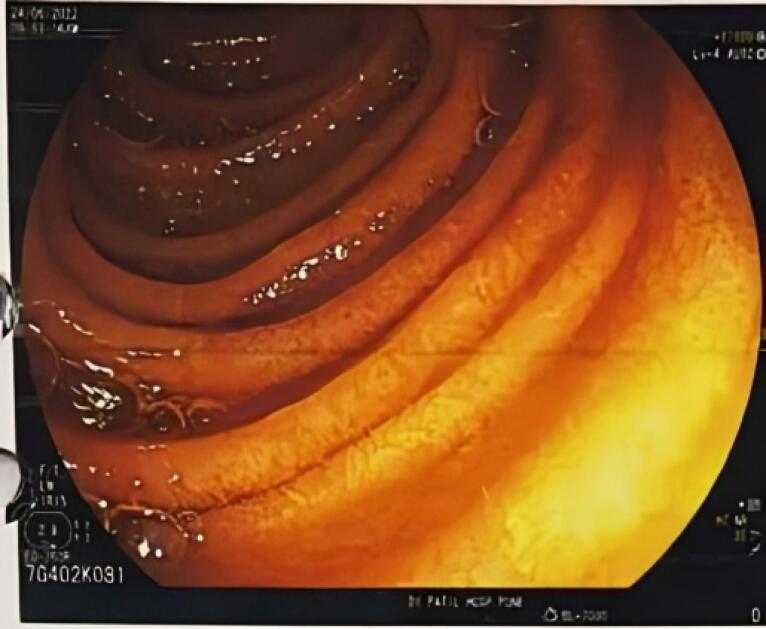
Colonoscopy image showing no other obvious abnormality

 The UGI endoscopy revealed edema in the esophagus and the colon (Figures 1 and 2). A histopathological specimen was taken and sent for examination. Urinary 24-hour protein was found to be 1427 mg/24 hours. This prompted us to also investigate the autoimmune panel, as the patient was a young female with subnephrotic proteinuria. The autoimmune panel showed the following results: C3 44.3 mg/dL (80-110), C4 15.5 mg/dL (10-40), antineutrophilic antibody blot: anti nRNP/Sm, anti Ro52, anti-mitochondrial antibody M2, anti-dsDNA and anti-Sm positive.

**Figure 3:**
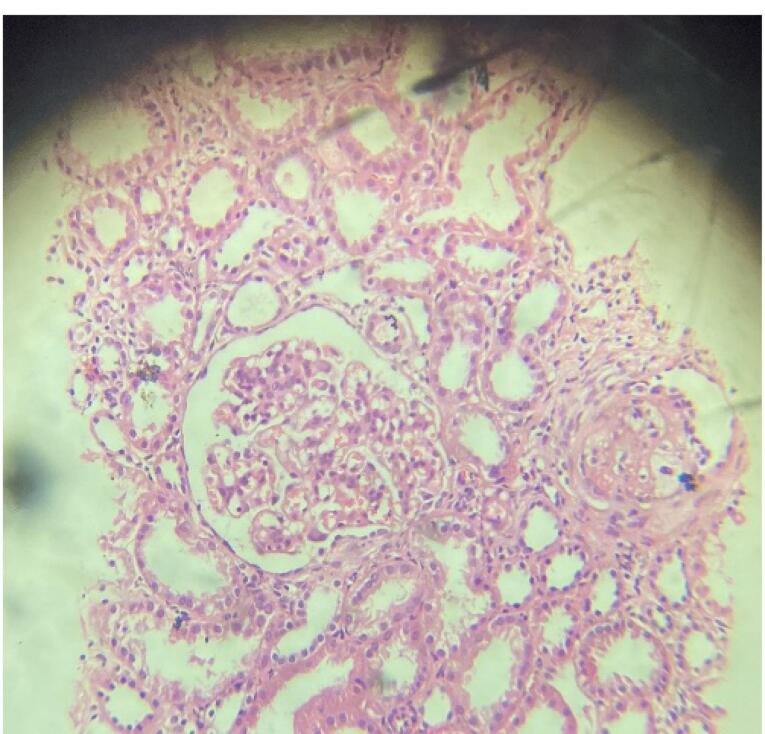
Renal biopsy image showing sclerosed glomerulus (Right) and a viable glomerulus (Left)

**Figure 4:**
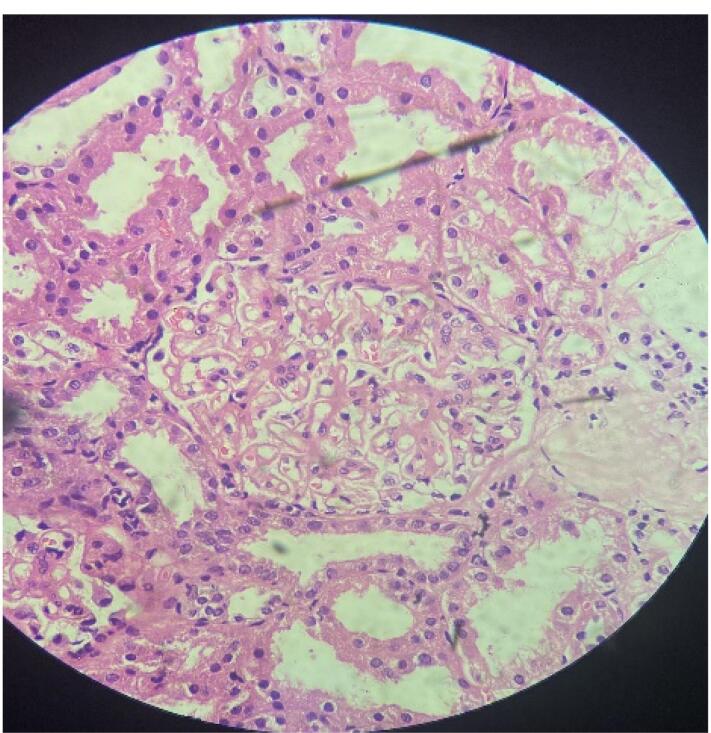
Renal biopsy image showingmesangial expansion, mesangial proliferation, some lobulation and focal sclerosis

 The UGI and colonoscopy histopathological biopsy specimen revealed eosinophilic infiltration of the bowel wall (Figures 1 and 2). Considering the autoimmune panel, a renal biopsy was planned for this patient. Renal biopsy showed: 26 glomeruli, one globally sclerosed (Figure 3), eight glomeruli showing mesangial proliferation (Figure 4) and lobulation. Tubules showed resorption droplets and tubulitis. Interstitium showing mononuclear infiltration. Blood vessels were unremarkable. Immunofluorescence showed positivity of IgG, IgM, IgA, C3 and C1q, the classical “full house” pattern. A diagnosis of LN class 3 (ISN-RPS) was made.

 The patient was initiated on pulse injectable methylprednisolone 500 mg for 3 days followed by oral prednisolone at 1 mg/kg and oral tablet hydroxychloroquine 200 mg/d. The patient was discharged. On follow-up after 4 weeks, she had complete relief of her abdominal symptoms, and the urine routine showed absent proteinuria with normal serum urea and creatinine.

## Discussion

 Lupus enteritis is a rare and poorly understood cause of abdominal pain in SLE. Clinical symptoms include abdominal pain (97%), vomiting (42%), diarrhea (32%), and fever (20%). Gastrointestinal symptoms are extremely distressing, but renal dysfunction remains the dreaded complication. Failure to treat aggressively may lead to end-stage renal disease in these patients.^3^ The kidney is the most commonly involved visceral organ in SLE. Although approximately 38% of patients may have renal dysfunction of some sort at presentation, with improved diagnostic and treatment modalities, death due to LN has reduced.^4^ Timely diagnosis remains of the essence. In women ages 15-24 years, SLE is the number one cause of death among chronic inflammatory diseases, ranking higher than diabetes mellitus.^5^ We put forth this case to bring clinicians’ attention to the possible variations and variety of this disease and to bear in mind the diagnosis of SLE in patients.
